# Estimated number of N95 respirators needed for healthcare workers in acute-care hospitals during the coronavirus disease 2019 (COVID-19) pandemic

**DOI:** 10.1017/ice.2020.1418

**Published:** 2021-01-11

**Authors:** Patrick T. Wedlock, Kelly J. O’Shea, Madellena Conte, Sarah M. Bartsch, Samuel L. Randall, Marie C. Ferguson, Sarah N. Cox, Sheryl S. Siegmund, Sarah Kulkarni, Denis Nash, Michael Y. Lin, Bruce Y. Lee

**Affiliations:** 1 Public Health Informatics, Computational, and Operations Research, CUNY Graduate School of Public Health and Health Policy, New York City, New York; 2 Institute for Implementation Science in Population Health, City University of New York, New York City, New York; 3 Division of Infectious Diseases, Department of Medicine, Rush University Medical Center, Chicago, Illinois

## Abstract

**Objective:**

Due to shortages of N95 respirators during the coronavirus disease 2019 (COVID-19) pandemic, it is necessary to estimate the number of N95s required for healthcare workers (HCWs) to inform manufacturing targets and resource allocation.

**Methods:**

We developed a model to determine the number of N95 respirators needed for HCWs both in a single acute-care hospital and the United States.

**Results:**

For an acute-care hospital with 400 all-cause monthly admissions, the number of N95 respirators needed to manage COVID-19 patients admitted during a month ranges from 113 (95% interpercentile range [IPR], 50–229) if 0.5% of admissions are COVID-19 patients to 22,101 (95% IPR, 5,904–25,881) if 100% of admissions are COVID-19 patients (assuming single use per respirator, and 10 encounters between HCWs and each COVID-19 patient per day). The number of N95s needed decreases to a range of 22 (95% IPR, 10–43) to 4,445 (95% IPR, 1,975–8,684) if each N95 is used for 5 patient encounters. Varying monthly all-cause admissions to 2,000 requires 6,645–13,404 respirators with a 60% COVID-19 admission prevalence, 10 HCW–patient encounters, and reusing N95s 5–10 times. Nationally, the number of N95 respirators needed over the course of the pandemic ranges from 86 million (95% IPR, 37.1–200.6 million) to 1.6 billion (95% IPR, 0.7–3.6 billion) as 5%–90% of the population is exposed (single-use). This number ranges from 17.4 million (95% IPR, 7.3–41 million) to 312.3 million (95% IPR, 131.5–737.3 million) using each respirator for 5 encounters.

**Conclusions:**

We quantified the number of N95 respirators needed for a given acute-care hospital and nationally during the COVID-19 pandemic under varying conditions.

Due to persistent and widespread shortages of N95 respirators during the ongoing coronavirus disease 2019 (COVID-19) pandemic in the United States, there is a need to estimate the number of N95 respirators that may be needed for frontline healthcare workers (HCWs) interacting with hospitalized COVID-19 patients. N95 respirators are a crucial component of infection control strategies that minimize HCW exposure to airborne particles and prevent droplets from coming into contact with the mouth and nose.^1^ However, many factors, including a dwindling national stockpile and a constrained manufacturing and supply chain capacity,^2-4^ have resulted in shortages of N95 respirators. Ensuring an adequate supply of respirators for frontline HCWs requires understanding the potential demand, which is dependent on how many people are exposed to COVID-19 coronavirus, how many of those exposed are subsequently hospitalized, and how long each patient remains in the hospital. In addition, varying respirator usage guidelines and patient encounters may alter how many respirators are ultimately needed.

Knowing the number of respirators needed in advance is essential for setting and achieving procurement targets and allocating respirators appropriately. For example, hospital administrators can plan ahead to order enough respirators to adequately cover all HCWs (and other essential personnel), while government officials and manufacturers can work together to ensure that production and financing of respirators is adequate to cover even the worst-case scenario. Although past research has estimated the potential demand for N95 respirators under certain assumptions,^5^ studies have not demonstrated how these estimates vary when different proportions of the population become exposed and under different operating conditions (eg, number of uses per respirator). Therefore, we developed a computational model to estimate how many N95 respirators would be needed both in a single acute-care hospital, varying with the number of COVID-19 admissions per month, and across the United States over the entire course of the pandemic when different proportions of the population are exposed to severe acute respiratory coronavirus 2 (SARS-CoV-2).

## Methods

### Single acute-care hospital model

Using Excel software (Microsoft, Redmond, WA) with the Crystal Ball add-in (Oracle, Redwood Shore, CA), we developed a computational model representing a single acute-care hospital to simulate the number of all-cause admissions and what can happen to each person who is hospitalized with confirmed or suspected SARS-CoV-2.^6,7^ For each simulation run, we first determined the number of confirmed and suspected COVID-19 admissions for a single acute-care hospital in a month. To determine the number of confirmed COVID-19 admissions in a month, we applied a COVID-19 prevalence among hospitalized patients to the number of all-cause admissions in that month. Each hospitalized COVID-19–positive patient then travels through a probability tree of different possible sequential clinical outcomes.^6,7^ First, the patient has a probability of having either severe pneumonia or severe nonpneumonia symptoms and has a probability of intensive care unit (ICU) admission. If admitted to the ICU, the patient has a probability of requiring a ventilator, representing acute respiratory distress syndrome (ARDS). The patient also has a probability of dying from COVID-19 complications. Each suspected COVID-19 patient who is later confirmed not to be infected (ie, COVID-19 negative), referred to as persons under investigation (PUI), remains under COVID-19 precautions for 1–2 days, accounting for test turnaround time. We assumed a ratio of 1–2 PUIs for every 10 patients hospitalized with COVID-19.

We assumed that N95 respirators are reserved for interactions between HCWs and hospitalized patients with a suspected or confirmed COVID-19 infection. Each hospitalized COVID-19 patient has 10 encounters per day with HCWs including doctors, nurses, and other staff for the duration of their hospital stay and can include repeated interactions with the same HCW multiple times. As hospitalized patients with more severe disease may require more frequent interactions with HCW, we ran additional scenarios in which patients in the ICU were visited more often per day than patients hospitalized in the general ward. HCWs dispose of an N95 respirator after one COVID-19 patient encounter, per N95 respirator manufacturer guidelines.^1^ These values vary in sensitivity analyses.

### US national model

We also developed a model representing the US population (327,167,434 persons) and what can happen to each person who gets infected with SARS-CoV-2 to determine the number of N95 respirators required for the entire United States over the course of the pandemic. This involved determining the percentage of the national population that becomes infected (ie, the attack rate) with the age distribution of cases matching the reported age distribution of COVID-19 cases. Each infected person then travels through a probability tree of different possible sequential clinical outcomes. First, the person has a probability of being asymptomatic throughout the entire course of the infection. If the person is symptomatic (ie, COVID-19 case), he or she starts with a mild infection and has a probability of seeking ambulatory care or calling a physician (ie, telephone consult). This person then has a probability of progressing to severe disease requiring hospitalization. If the person is hospitalized, he or she travels through the clinical progression of hospitalized patients as described above. The number of N95 respirators needed are calculated as described above, but for all COVID-19 hospital admissions.

### Data sources

Table 1 shows key model input parameters, values, and sources. All clinical probabilities and durations are age-specific when available and come from scientific literature or nationally representative data sources. Age-specific COVID-19 data are specific to the US context as of May 30, 2020.^8^

**Table 1.** Model Input Parameters, Values, and Sources

Parameter	Distribution Type	Mean^a^ or Median	Standard Error or Range	Source
**Probabilities**				
Asymptomatic infection	Triangular	0.35	0.315–0.385^b^	CDC^21^
Relative infectiousness of asymptomatic infection	Point estimate	1	…	CDC^21^
Missing work/school	Point estimate	1.0	…	Assumption
**Ambulatory care**				
0–4 y	Beta	0.455^a^	0.098	Molinari et al^22^
5–17 y	Beta	0.318^a^	0.061	Molinari et al^22^
18–64 y	Beta	0.313^a^	0.014	Molinari et al^22^
≥65 y	Beta	0.62^a^	0.027	Molinari et al^22^
**Probability of hospitalization, given infection**				
0–17 years old	Beta Pert	0.031	0.028-0.034^b^	Stokes et al^23^
18–44 years old	Beta Pert	0.056	0.051-0.062^b^	Stokes et al^23^
45–64 years old	Beta Pert	0.140	0.126-0.154^b^	Stokes et al^23^
≥65 years old	Beta Pert	0.300	0.270-0.330^b^	Stokes et al^23^
**Probability of ICU admission**				
0–17 y	Beta Pert	0.171	0.154-0.1881^b^	Stokes et al^23^
18–44 y	Beta Pert	0.152	0.137-0.1675^b^	Stokes et al^23^
45–64 y	Beta Pert	0.186	0.168-0.2047^b^	Stokes et al^23^
≥65 y	Beta Pert	0.148	0.133-0.163^b^	Stokes et al^23^
**Probability of mortality**				
0–17 y	Beta Pert	0.022	0.019-0.024^b^	Stokes et al^23^
18–44 y	Beta Pert	0.075	0.067-0.082^b^	Stokes et al^23^
45–64 y	Beta Pert	0.208	0.187-0.228^b^	Stokes et al^23^
≥65 y	Beta Pert	0.607	0.546-0.668^b^	Stokes et al^23^
Pneumonia, given hospitalization	Beta	0.79	0.711-0.869^c^	Guan et al^24^
ARDS, requiring ventilator use in ICU	Beta	0.771^a^	0.053	Arentz et al^25^ Richardson et al^26^ Gold et al^27^ Bhatraju et al^28^ Auld^29^
**Age group, given infection**				
0–17 y	Point estimate	0.05	…	Stokes et al^23^
18–44 y	Point estimate	0.39	…	Stokes et al^23^
45–64 y	Point estimate	0.33	…	Stokes et al^23^
≥65 y	Point estimate	0.23	…	Stokes et al^23^
**Durations, d**				
Ambulatory care	Point estimate	0.5	…	Assumption
Duration of symptoms with mild illness	Triangular	7	3–17	Arashito et al^30^ Duszynski et al^31^ Chang et al^32^
Duration of symptoms prior to hospital admission	Triangular	7	3–9^d^	Garg et al^33^ Wang et al^34^
**Hospitalization, not admitted to ICU, age group**				
0–49 y	Gamma	3.9^a^	3.7	CDC^21^
50–64 y	Gamma	4.9^a^	4.3	CDC^21^
≥65 y	Gamma	6.2^a^	5.7	CDC^21^
Hospitalization, ICU (all ages)	Gamma	9	4–17^c^	Gold et al^27^ Bhatraju et al^28^ Auld et al^29^ Lewnard et al^35^
Hospitalization, ventilator use	Gamma	9	5–12^c^	Gold et al^27^ Bhatraju et al^28^ Imam et al^36^

Note. CDC, Centers for Disease Control and Prevention; ICU, intensive care unit; ARDS, acute respiratory distress syndrome.

a Mean.

b Values are ±10% of median value.

c Values are interquartile range.

d Values are 10%–90%.

### Scenarios and sensitivity analyses

Experiments consisted of Monte Carlo simulations of 2,000 trials, varying parameters throughout their range (Table 1). The first set of experiments simulated what may happen to a hospital as the COVID-19 prevalence among patient admissions increases from 0.5% to 100%, varying with the number of all-cause hospital admissions per month (50–2,000). The second set of experiments simulated what may happen nationally over the course of a pandemic as the attack rate increases from 5% to 90%. Different scenarios represent what may happen under varying respirator usage guidelines, that is, the number of patient encounters for which a given respirator can be used (1–20), and when varying the number of encounters each patient has with HCW and staff over the course of 24 hours (total for all staff, 5–20).^9^ We also varied the frequency at which HCWs interact with ICU patients compared to general ward patients (2–4 times more frequently). Sensitivity analyses varied key parameters including the distributions of patients’ length of stay (eg, ±50% current values), and the probability of different COVID-19 outcomes (eg, probability of hospitalization, ICU admission, and ventilator use). We report results as the median and the 95% interpercentile range (IPR) from 2.5 to 97.5.

## Results

### N95 respirators needed for COVID-19 admissions per month in a single acute-care hospital

**Fig. 1.**
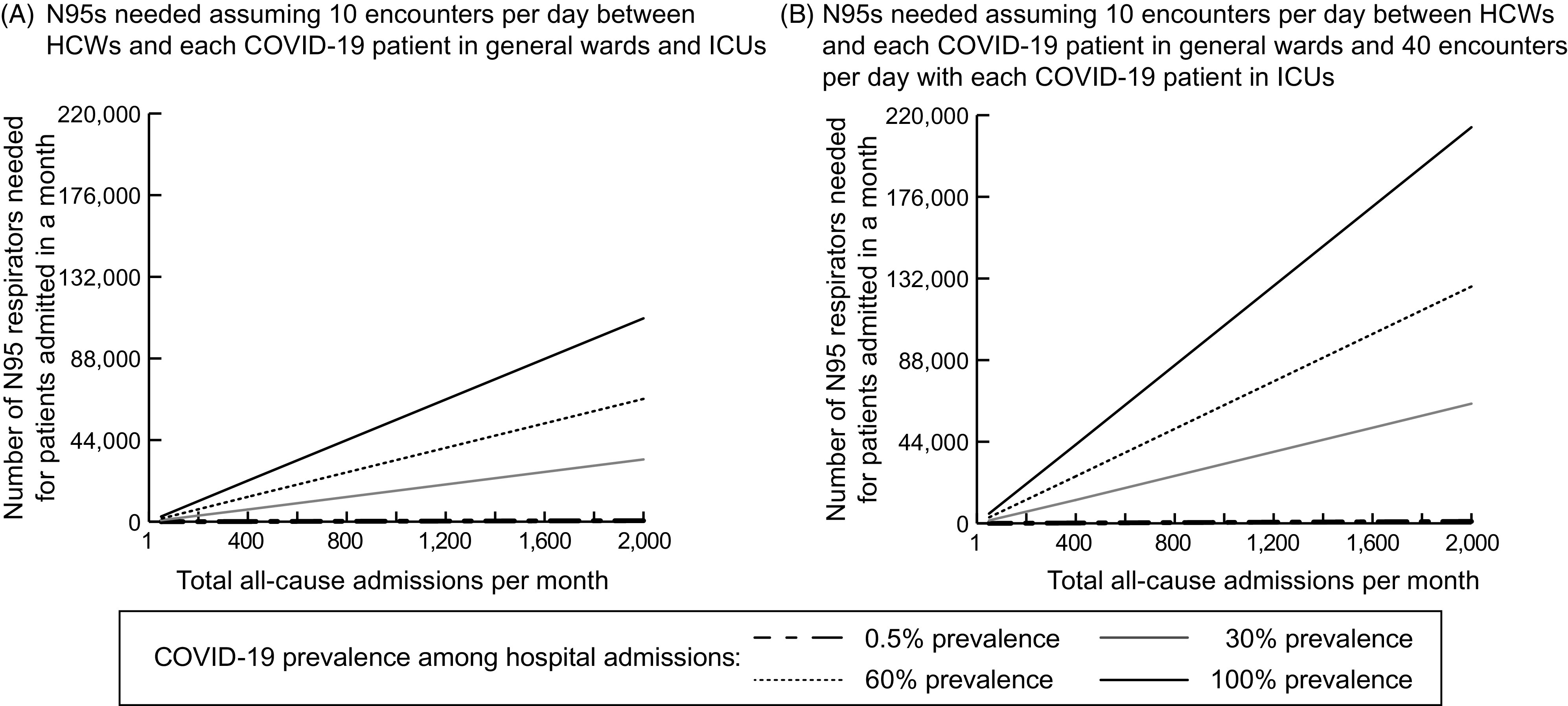
Number of N95 respirators needed in an acute-care hospital in the United States as the prevalence of COVID-19 among hospital admissions increases when there are (A) 10 encounters per day between healthcare workers (HCWs) and COVID-19 patients in general wards and ICUs and when there are (B) 10 encounters per day between HCWs and COVID-19 patients in general wards and 40 encounters per day with COVID-19 patients in ICUs.

Figure 1 shows how changes in the prevalence of COVID-19 among hospital admissions per month influences the number of N95 respirators needed for a single acute-care hospital. For example, in a hospital with 50 all-cause admissions per month, the number of respirators needed for COVID-19 admissions that month (ie, all confirmed COVID-19 patients and PUI) ranges from 820 (95% IPR, 381–1,674) to 1,667 (95% IPR, 798–3,498) with a prevalence of COVID-19 among admissions of 30% to 60%, assuming respirators are disposed after each use and there are 10 encounters per day between HCWs and COVID-19 patients. A hospital averaging 400 all-cause admissions per month would require 6,580 (95% IPR, 3,033–12,918) to 13,378 (95% IPR, 5,904–25,881) respirators for the COVID-19 patients admitted that month (30%–60% COVID-19 admission prevalence). Varying the frequency at which HCWs interact with COVID-19 patients in the ICU up to 4 times more compared to patients in the general ward (ie, 10 encounters between HCWs and COVID-19 patients in the general ward vs 40 encounters per day in ICU) increases the number of respirators required to 12,593 (95% IPR, 6,429–22,725) to 25,327 (95% IPR, 12,923–44,951) as COVID-19 prevalence increases from 30% to 60% in a hospital with 400 all-cause admissions per month (single-use N95).

**Fig. 2.**
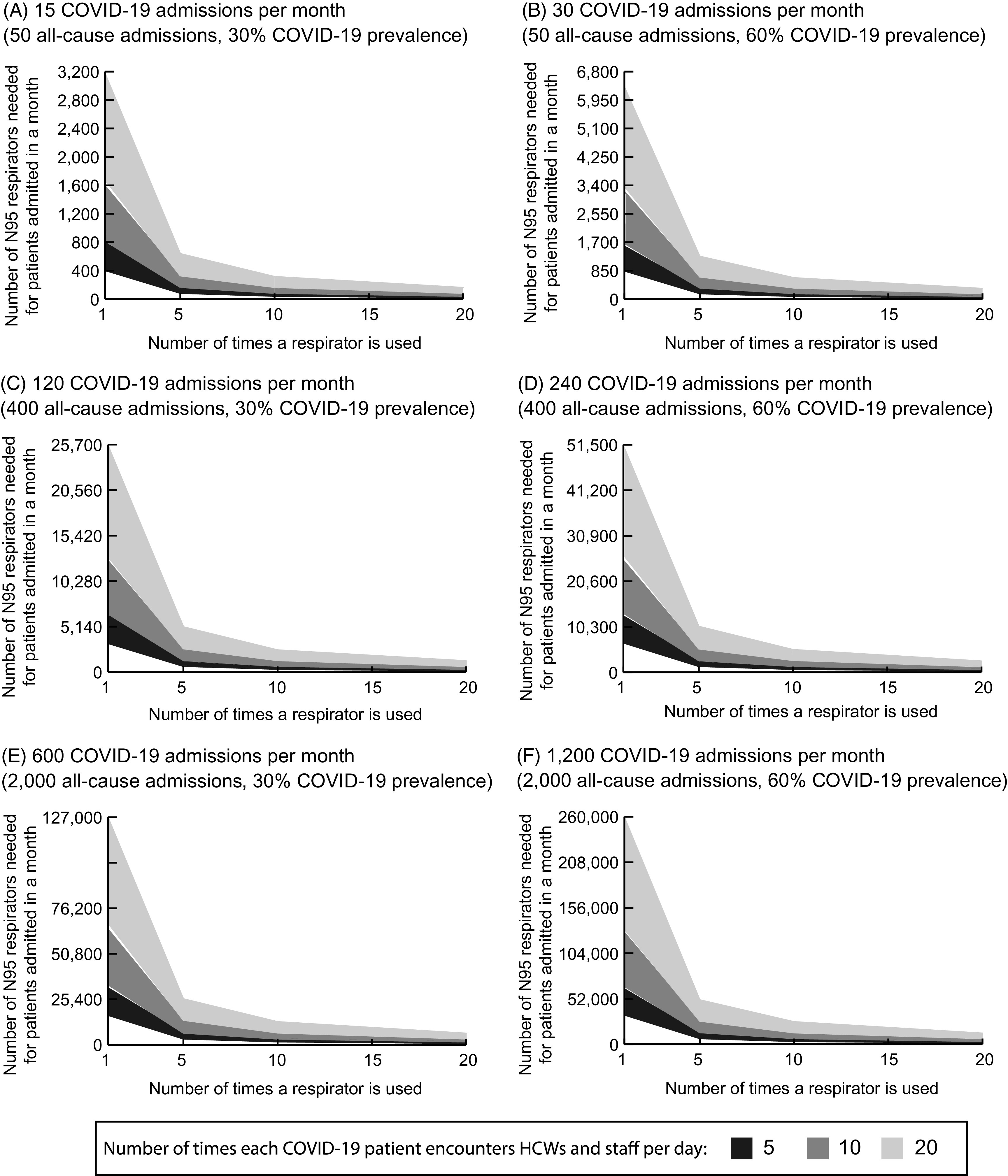
Number of N95 respirators needed in an acute-care hospital when varying respirator use guidelines, increasing the number of patient encounters for which a given respirator can be used for various example number of COVID-19 patient admissions per month (ie, for various all-cause admissions and COVID-19 prevalence values). The lower bound of the band represents the minimum number needed when healthcare worker (HCW)–patient interactions are the same for COVID-19 patients in the general ward and ICU. The upper bound represents the maximum number needed when HCW–patient interactions are 4 times higher for ICU patients compared to general ward patients. Note difference in scales across panels.

Figure 2 illustrates how increasing the number of times an N95 respirator is used before discarding reduces the number of respirators a hospital needs for COVID-19 patients. For example, a hospital with 400 all-cause monthly admissions with a 30% COVID-19 admission prevalence (ie, 120 COVID-19 admissions per month) and 10 encounters per day between HCWs and COVID-19 patients a hospital would require 1,320 (95% IPR, 600–2,693) N95s if reusing an N95 respirator 5 times (2,548; 95% IPR, 1,280–4,337 when encounters are 4 times as frequent in the ICU versus a general ward). When respirators can be reused between 10 and 20 times (which can translate to 1–2 days), a hospital would require 337 N95s (95% IPR, 156–660) to 669 N95s (95% IPR, 308–1,309), or 635 N95s (95% IPR, 330–1,095) to 1,286 N95s (95% IPR, 644–2,299) when encounters are 4 times more frequent for patients in the ICU. As another example, in a hospital with 2,000 monthly all-cause admissions, reusing N95 respirators 5–10 times would require 6,645 respirators (95% IPR, 3,061–13,591) to 13,404 respirators (95% IPR, 6,106–26,116) with a 60% COVID-19 admission prevalence (ie, 1,200 COVID-19 admissions per month) and 10 daily encounters between HCWs and a COVID-19 patient. For the same hospital, this number increases to 12,739 respirators (95% IPR, 6,582–22,353) to 25,492 respirators (95% IPR, 13,073–45,781) when encounters are 4 times more frequent for ICU patients.

Figure 2 also shows the impact of varying the number of interactions between HCWs and each COVID-19 patient on the total number of N95s needed. For example, a hospital with 400 admissions per month with a 30% COVID-19 admission prevalence (120 monthly COVID-19 admissions) would require 13,026 respirators (95% IPR, 6,524-25,568) with 20 interactions per day (25,611; 95% IPR, 12,607–44,117 when ICU visits are 4 times more frequent) and 3,255 respirators (95% IPR, 1,559–6,572) with 5 interactions per day (6,416; 95% IPR, 3,188–11,229 when ICU visits are 4 times more frequent), assuming that each N95 is used once before being discarded.

### N95 respirators needed for COVID-19 admissions nationally over the course of the pandemic

Table 2 shows how the total number of N95 respirators needed changes as the COVID-19 pandemic progresses and more people get infected (ie, as the attack rate increases). For example, if 20% of the US population become infected with SARS-CoV-2 (ie, 20% attack rate) over the course of the pandemic, there would be a median of 42.5 million (95% IPR, 40.9–44.1 million) symptomatic COVID-19 cases and 33.1 million (95% IPR, 13.5–79.0 million) hospital bed days. Assuming single-use N95 respirators and 10 HCW-patient encounters per day, the United States would require a total of 343.8 million (95% IPR, 145.5–806.7 million) N95 respirators. At a higher attack rate (50%), there would be 106.3 million (95% IPR, 102.3–110.3 million) symptomatic cases and 82.8 million (95% IPR, 33.7–197.5 million) hospital bed days, requiring 860 million (95% IPR, 370.7 million–2.0 billion) N95s in the United States under single-use guidelines.

**Table 2.** Clinical Outcomes and N95 Respirators (in Millions) Needed Nationally Across Different Attack Rates^a^

Outcome	Attack Rate			
	5%	20%	50%	90%
Symptomatic cases	10.6 (10.2–11.0)	42.5 (40.9–44.1)	106.3 (102.3–110.3)	191.4 (184.1–198.5)
Hospitalizations	1.5 (1.4–1.6)	5.9 (5.5–6.3)	14.7 (13.9–15.7)	26.5 (25.0–28.2)
GW bed days	6.1 (1.6–16.9)	24.2 (6.3–67.5)	60.6 (15.7–168.9)	109.1 (28.3–303.9)
ICU bed days	2.3 (0.9–4.9)	9.0 (3.8–19.8)	22.6 (9.4–49.4)	40.6 (16.9–89.0)
Total hospital bed days	8.3 (3.4–19.8)	33.1 (13.5–79.0)	82.8 (33.7–197.5)	149.1 (60.6–355.5)
**N95 respirators needed when HCWs interact with COVID-19 ICU patients at different rates than GW patients**				
10 encounters for GW patients and 10 encounters for ICU patients	86.0 (37.1–200.6)	343.8 (145.5–806.7)	860.0 (370.7–2,006.0)	1,548.0 (667.3–3,610.8)
10 encounters for GW patients and 20 for ICU patients	112.0 (53.2–228.7)	447.7 (213.8–921.6)	1,119.7 (532.1–2,286.8)	2,015.4 (957.8–4,116.2)
10 encounters for GW patients and 40 for ICU patients	160.5 (80.2–295.7)	642.6 (321.7–1,186.9)	1,605.2 (802.5–2,956.5)	2,889.4 (1,444.5–5,321.7)

Note. GW, general ward; ICU, intensive care unit; HCW, healthcare worker.

a Assumes 10 healthcare worker to patient encounters per day and that each N95 respirator is used only once.

**Fig. 3.**
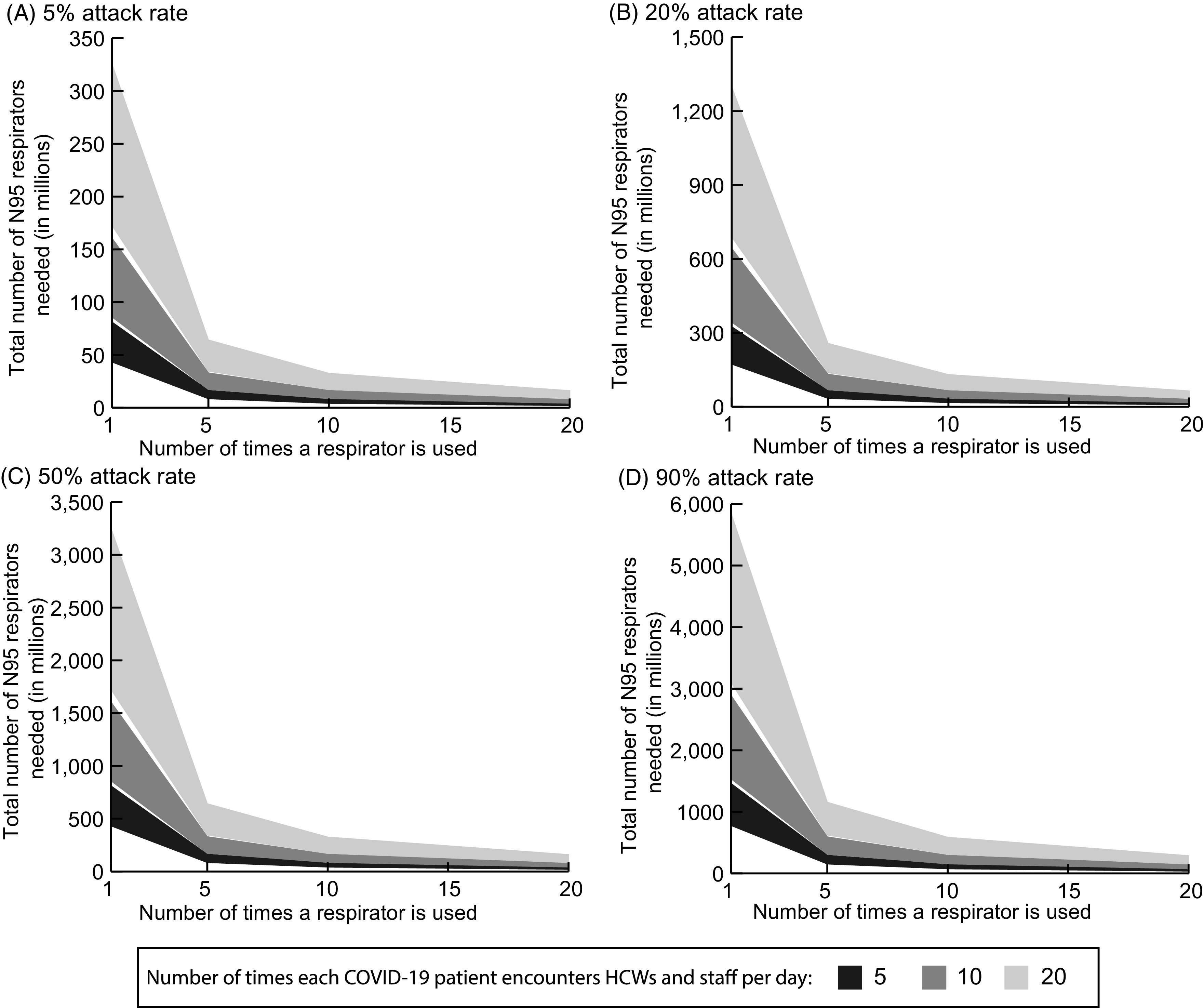
Total number of N95 respirators needed nationally in the United States when varying respirator use guidelines, increasing the number of patient encounters for which a given respirator can be used for various example attack rates (ie, when various proportions of the population get infected with COVID-19 coronavirus) over the course of the pandemic. The lower bound of the band represents the minimum number needed when healthcare worker (HCW)–patient interactions are the same for the general ward and ICU. The upper bound represents the maximum number needed when HCW–patient interactions are 4 times the number for ICU patients compared to general ward patients. Note difference in scales across panels.

Figure 3 shows how varying the number of N95 respirator uses and encounters between HCWs and each COVID-19 patient influences the number of N95 respirators needed and how this varies with attack rate. For example, allowing respirators to be used for 5 patient interactions before being discarded would require 17.4 million respirators at a 5% attack rate, assuming that HCWs have 10 encounters per day with each COVID-19 patient, and 173.5 million respirators at a 50% attack rate. Figure 3 also shows how the number of encounters influences the number of respirators needed during the course of the pandemic and how this varies with attack rate. The number of N95 respirators needed increases linearly with increases in the number of encounters per day. For example, when each N95 is used for only 1 encounter, if HCWs have 20 encounters with a COVID-19 patient per day (compared to 10), the number of N95 respirators needed nationwide is 172.7 million (95% IPR, 74.6–405.7 million) with a 5% attack rate and 1.7 billion (95% IPR, 0.7–4.1 billion) with a 50% attack rate.

The number of respirators needed also varies with healthcare usage (eg, varying length of stay and the probability of health outcomes resulting in hospitalization). For example, decreasing the probability of hospitalization and ICU admission by a relative 50% results in 158.2 million (95% IPR, 60.6–379.6 million) total N95 respirators needed, while increasing these probabilities by a relative 50% increases the number of total needed to 565.4 million (95% IPR, 261.2 million–1.2 billion) when N95 respirators are used for 1 encounter each (20% attack rate; 10 HCW–patient encounters per day). When decreasing or increasing hospital length of stay by a relative 50%, the number of a respirators needed ranges from 180.0 million (95% IPR, 81.3–397.2 million) to 510.3 million (95% IPR, 214.1 million–1.2 billion) when N95 respirators are used for 1 encounter each before discarding (20% attack rate; 10 HCW–patient encounters per day).

## Discussion

Our results demonstrate that there are many conditions under which a single acute-care hospital may require thousands of N95 respirators for HCWs to manage COVID-19 patients admitted in a given month. For hospitals with 400–2,000 all-cause admissions per month (which tend to be mid-to-larger hospitals in size or those with high patient turn over) with a high prevalence of COVID-19 among admissions (eg, 60%), the number of N95 respirators needed could be in the tens of thousands for COVID-19 patients admitted in a given month. Nationwide, if the COVID-19 pandemic infects 20%–50% of the United States population, the total number of N95 respirators needed to manage hospitalized COVID-19 patients could range from 170 million to >3 billion, under the most conservative N95 usage guidelines (ie, single use before disposal). As the COVID-19 pandemic continues to spread, results can help individual hospitals, state and federal government officials, and manufacturers plan for a wide variety of potential scenarios.

At the hospital level, our results provide an estimate for hospital decision makers (eg, hospital administrators, infection control) to understand their potential excess demand of N95 respirators due to COVID-19, which can be used to both plan for procurement and to identify potential preservation strategies in the event of a shortage. Although the size of the hospital (represented by number of all-cause admissions) and the number of COVID-19 admissions is an important driver of the potential N95 respirators needed, even a single acute-care hospital with 50 all-cause admissions per month could require >1,000 respirators for COVID-19 patients in a month when the prevalence of COVID-19 among hospitalized patients is high (eg, >30%). As such, it is important for hospitals of all sizes to be prepared for these scenarios.

Additionally, our results show how certain strategies could reduce the N95 need for hospitals, which could be particularly important as more and more of the population becomes infected. Strategies might include setting a limit of 5 reuses per respirator, adopting extended use practices, wearing a surgical mask over a respirator, or reducing the frequency of HCW–patient interactions. Based on manufacturers’ guidelines, N95 respirators are intended to be used once, properly disposed of, and replaced with a new respirator.^1,10^ Some data suggest, however, that HCWs could safely reuse respirators for up to 5 encounters,^11^ while extended-use policies suggest that an N95 can be worn for at least a few hours.^12^ Although the Centers for Disease Control and Prevention (CDC) and the National Institute for Occupational Safety and Health (NIOSH) do not specifically recommend respirator reuse, they acknowledge these strategies are options in times of scarcity and provide guidance for those circumstances.^10,13^ Hospitals could take steps to facilitate reuse of existing N95 respirators by adopting sterile reprocessing of the respirators (eg, hydrogen peroxide decontamination); however, such approaches are costly, are not standardized, and require strict compliance to implement effectively.^14,15^

At the national level, our study has demonstrated that respirator requirements should be more coordinated than they have been thus far in the pandemic. Although the number of N95 respirators used to date during the pandemic in the United States is unclear, it is estimated that the demand is 140 million during a 90-day peak-use period,^16^ though this demand likely reflects use beyond the acute-care hospital setting. In light of increasing COVID-19 infections, and reports from 3M that the demand is more than the entire industry can supply for the foreseeable future,^17^ it is critical to improve the supply of these respirators, potentially by broadening the use of the Defense Production Act as supported by the American Medical Association, American Hospital Association, and American Nurses Association.^18^ By anticipating the potential magnitude of demand for N95s and how this demand can be affected by different epidemiological circumstances and policies, governments and hospitals can better prepare to meet the demands of this pandemic and future outbreaks.

This study has several limitations. All models, by definition, are simplifications of real life and cannot account for every outcome.^19^ Model inputs drew from various sources, and new data on COVID-19 continually emerge. Sensitivity analyses helped determine the impact of uncertainty and variability in the available data. Estimates of the number of patients that are seen per HCW can vary by healthcare facility and circumstance. Additionally, determining when a patient encounter begins and ends is difficult (eg, entering a COVID-19 ward vs caring for a particular patient), which can make estimating the number of respirators needed challenging. Furthermore, this study only accounted for N95 respirator use for hospitalized patients and did not include outpatient settings or congregate settings (eg, nursing homes) because recommendations for N95 respirator usage vary outside intensive care settings with alternative options available. Furthermore, although some hospitals may use different types of respirators (eg, N100s, elastomeric respirators), the extent to which these are used is unclear. As such, our analysis focused on the use of N95 respirators. We also focused on the total number of respirators needed at different attack rates regardless of the duration of the outbreak, which would have implications for manufacturing capacity. Finally, our model does not account for the possibility of a single patient becoming hospitalized again for the same COVID-19 infection. Recent CDC estimates indicate ∼9% of hospitalized patients are readmitted.^20^

In conclusion, our study quantifies the number of N95 respirators needed for a single acute-care hospital and nationally during the COVID-19 pandemic under varying conditions.
